# Sex-Specific Growth Rates of Ascending Thoracic Aortic Aneurysms in Non-Syndromic Patients: A Systematic Review

**DOI:** 10.3390/diagnostics16060916

**Published:** 2026-03-19

**Authors:** Rebecca M. J. Gylling, Heidi M. Pokka, Oke Gerke, Joachim S. Skovbo, Jes S. Lindholt, Axel C. P. Diederichsen, Sebrina M. Hansen, Lasse M. Obel

**Affiliations:** 1 Department of Nuclear Medicine, Odense University Hospital, 5000 Odense, Denmarkoke.gerke@rsyd.dk (O.G.); 2 Department of Clinical Research, University of Southern Denmark, 5000 Odense, Denmark; 3 Elite Centre for Individualized Medicine in Arterial Disease (CIMA), Odense University Hospital, 5000 Odense, Denmark; lasse.mollegaard.obel@rsyd.dk; 4 Department of Cardiothoracic and Vascular Surgery, Odense University Hospital, 5000 Odense, Denmark; 5 Department of Cardiology, Odense University Hospital, 5000 Odense, Denmark; 6 Research Unit OPEN, University of Southern Denmark, 5000 Odense, Denmark; 7 Department of Biochemistry and Immunology, Lillebælt Hospital, University Hospital of Southern Denmark, 7100 Vejle, Denmark

**Keywords:** aortic growth rate, ascending thoracic aortic aneurysm, dilatation of ascending aorta, non-syndromic patients, sex differences, systematic review

## Abstract

**Background/Objectives**: Ascending thoracic aortic aneurysms (aTAAs) pose a high risk of dissection and rupture. Though more prevalent in males, females may experience worse outcomes. Growth rate is considered a part of risk assessment, yet data in non-syndromic females without valve abnormalities remain limited. This study aims to assess whether aTAA growth differs between non-syndromic females and males with normal aortic valve morphology. **Methods**: The systematic review followed the PRISMA 2020 guideline. The final search was completed in April 2025, with guidance from a certified librarian. Included studies were RCTs or observational studies of non-syndromic adults with aTAA reporting sex-specific data and included ≥10 females. Prior dissection, valve replacement, or surgery were excluded. In addition to the original search, 11 articles were identified as likely to contain sex-specific data, and the corresponding authors were contacted. The protocol is registered in PROSPERO (CRD420251025890). Meta-analysis was not performed due to high heterogeneity and limited study numbers. **Results**: Of 2629 identified studies, 73 studies were screened in full-text, and only three met the inclusion criteria. The most common exclusion reason was lack of appropriately sex-stratified data. Two authors out of the 11 contacted replied with additional datasets, resulting in a total of five studies being included. Of the five included studies, three found faster growth rates in females. Reported growth rates in females varied notably, ranging from −0.7–1.74 mm/year. **Conclusions**: Evidence on sex differences in aTAA growth among non-syndromic patients with normal aortic valves remains inconclusive. Three of the five studies reported faster growth in females. Standardization in future research is needed.

## 1. Introduction

**Figure 1:**
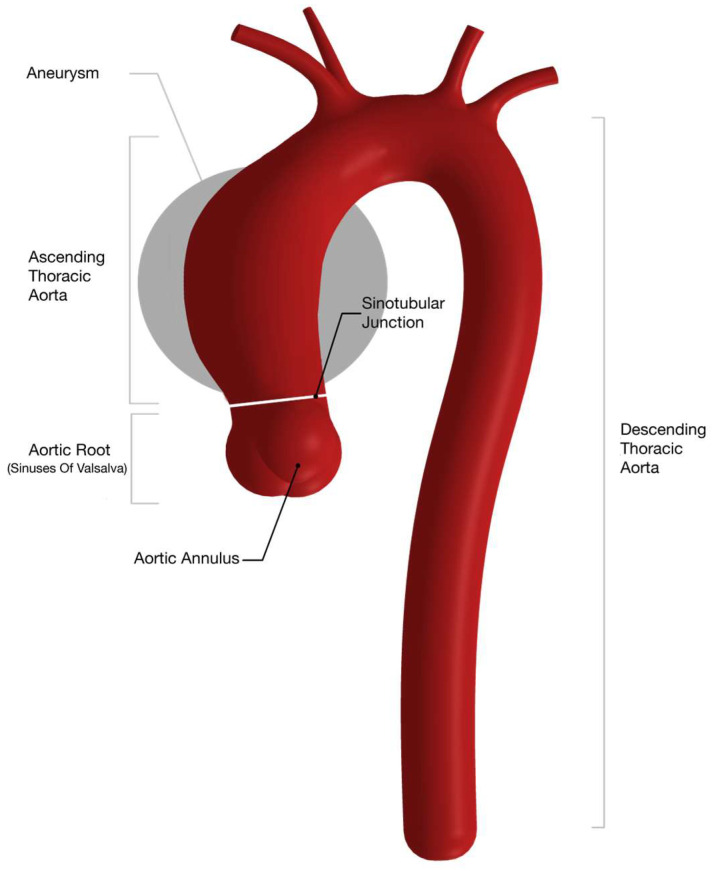
Thoracic aorta with marked segmentation relevant for imaging assessment of diameter growth. Aneurysm site of interest reviewed in this article circled in gray, corresponding to tubular ascending or mid-ascending segment of aorta.

Ascending thoracic aortic aneurysms (aTAAs) are the most common type of thoracic aortic aneurysm [1,2]. Aortic aneurysms are associated with a high risk of comorbidity and death when they occasionally rupture or dissect [3,4]. The risk of such adverse aortic events is suggested to increase with larger aortic diameters, as noted by Cozijnsen et al. [5]. Various risk factors have been identified, including sex differences [6]. Figure 1 illustrates segmentations of the ascending thoracic aorta relevant for the imaging assessment of an aneurysm growth.

Although aTAAs are more prevalent in males, a study from over two decades ago reported higher rates of adverse outcomes, including mortality, in females [7]. More recent studies have supported this finding [8]. However, evidence on sex differences in aTAA growth rates remains inconsistent.

Growth rate is central to risk prediction and the management of dissection or rupture [1,9]. Some studies, such as the DisSEXion study by Notenboom et al., report significantly higher growth rates in females, while others find no difference [6,10]. A faster growth rate could partly explain the higher risk of adverse outcomes observed in females [11,12]. However, interpretation of absolute growth rates is complicated by considerable methodological heterogeneity across studies, including differences in imaging modality, measurement protocols, and cohort composition [13].

In addition to biomechanical and anatomical determinants, ascending aortic aneurysm progression is increasingly recognized as a biologically active process involving chronic inflammation, immune activation, and extracellular matrix remodeling [14,15]. These processes may influence aortic wall integrity and disease progression and could contribute to inter-individual variability in growth behavior, including potential sex-specific differences. Furthermore, ascending thoracic aortic disease is frequently associated with congenital or hereditary factors, both of which comprise a genetically heterogeneous group of disorders; however, this systematic review focuses exclusively on non-syndromic patients to establish sex-specific growth rates in this distinct clinical cohort [9].

Although the DisSEXion study was rigorously conducted and provided important insights, it did not stratify growth rates by sex in relation to syndromic status or valve morphology. Likewise, recent systematic reviews—including Henry et al. (2025) and Cozijnsen et al. (2024)—have highlighted heterogeneity across aTAA imaging studies [5,13]. Taken together, these inconsistencies underscore the need for the careful synthesis of available evidence while acknowledging that existing studies are not methodologically superimposable.

The aim of this study was to determine whether female sex is associated with faster aTAA growth compared to male sex. This systematic review focused exclusively on non-syndromic patients and excluded individuals with bicuspid aortic valves (BAV), as current ESC and AHA clinical guidelines on the management of aortic disease already apply distinct risk stratification and management recommendations for these conditions [1,9].

## 2. Materials and Methods

This systematic review followed the 2020 Preferred Reporting Items for Systematic Review Checklist (PRISMA) [16], and the completed PRISMA 2020 Abstracts and expanded checklists are provided in Supplementary Materials Section S3. A preregistered protocol was published in PROSPERO for transparency and can be found via https://www.crd.york.ac.uk/PROSPERO/view/CRD420251025890 (accessed on 4 April 2025).

The methodological quality and the risk of the bias of the included studies were assessed using a customized Quality of Studies (QoS) evaluation tool. The QoS score of each study was considered when interpreting the evidence and drawing conclusions.

### 2.1. Systematic Literature Search

The five following databases were used in the search: EMBASE and MEDLINE via the Ovid platform, PubMed, Elsevier Scopus and the Cochrane Central Register of Controlled Trials. Despite existing overlap between databases, all were included to ensure comprehensive and highly sensitive search. To optimize the search strategy, a certified librarian (SMH) at the University of Southern Denmark was consulted for advice, while the search string development and execution were performed by the authors H.M.P. and R.M.J.G. There were no restrictions on the publication date. The final search was made in April 2025. A complete list of the search terms used can be found under Supplementary Materials (S1). In addition to the initial search, reference and citation searches were conducted using the articles included and the relevant recent reviews.

The eligibility criteria for this review required the inclusion of original research studies conducted as randomized controlled trials, prospective or historical cohort studies, or longitudinal follow-up studies. Studies were eligible if they were performed in non-syndromic adult patients (>18 years of age) with an ascending thoracic aortic aneurysm and without prior aortic dissection or rupture, valve replacement, or aortic surgery.

Studies were excluded if the reporting of growth rates was not sex stratified, if they included syndromic ascending thoracic aortic aneurysms or patients with congenital heart disease, or if the female sample size was fewer than 10. In addition, studies including both bicuspid and tricuspid aortic valves were eligible only if the analysis and reporting of aortic growth rates were stratified by valve morphology and sex.

The review process was conducted by two authors (HMP and RMJG) using Covidence (Veritas Health Innovation, Melbourne, Australia). Duplicate records were automatically identified in Covidence. Titles, abstracts, and full texts were screened independently by both reviewers, and any conflicts were resolved through discussion and consultation with a senior author (OG).

### 2.2. Data Extraction

Data extraction from the included studies was performed independently by HMP and RMJG. EndNote (Clarivate Analytics, Philadelphia, PA, USA) was used for reference management. The baseline characteristics chosen for extraction were based on recent studies highlighting the multifactorial background of aneurysm formation [17,18]. Absolute growth rates for females, males, and the total study population were extracted together with the *p*-values for the difference between sexes. Where growth rates for the ascending aorta were reported by subsegment, the data for the tubular ascending or mid-ascending aorta were preferred for extraction. All relevant literature reported growth rates in mm/year. Data extraction and synthesis were done using Microsoft Excel (Microsoft 365, Version 2511), and the data was noted using tables. In addition to the three studies included through the review process, 11 additional relevant studies were identified from the search results, and the corresponding authors were contacted. See Supplementary Materials Section S1, Table S1.4.1 for a comprehensive list of excluded studies, including the 11 studies which are in bold text. This process resulted in the additional inclusion of two datasets.

### 2.3. Quality Assessment

A customized Quality of Studies (QoS) Evaluation Tool was developed to assess methodological quality, such as imaging and reporting standards, and risk of bias (RoB) of the studies included. Preexisting quality evaluation tools were used as inspiration [19], but none met the specific methodological aspects related to imaging studies evaluating aortic aneurysm growth rates. The QoS tool consists of seven evaluation items, of which item 1–4 addresses methodological quality and items 5–7 evaluate the RoB. Each item was scored on a scale from 0 to 1, allowing half-points for five items, with a total score from 0 to 7. The total score placed the study in one of the following three categories: poor (0–2.5), moderate (3.0–5.0), and good (5.5–7.0). The score reflects the reliability of the results and reproducibility of the study. A more comprehensive guide to the QoS tool can be found in Supplementary Materials, Section S2 where Table S2.1 provides a detailed guide to grading.

### 2.4. Statistical Analysis

Descriptive statistics comprised mean ± standard deviation for continuous and frequency tables for categorical variables. We displayed aTAA growth rate with a Forest plot, without attempting to pool estimates due to large inter-study variation. When individual patient data was available [20,21], we estimated the median growth rate and supplemented a bootstrapped 95% confidence interval (95% CI) to replace mean growth and a respective Wald-type 95% CI in order to report more robust estimates. Given the limited number of eligible studies and substantial methodological heterogeneity, no pooled meta-analysis or meta-regression analyses were planned or performed. We used STATA Now/BE (Version 19.5, StataCorp LLC, College Station, TX, USA) for all calculations.

## 3. Results

### 3.1. Selection of Studies and Study Characteristics

**Figure 2:**
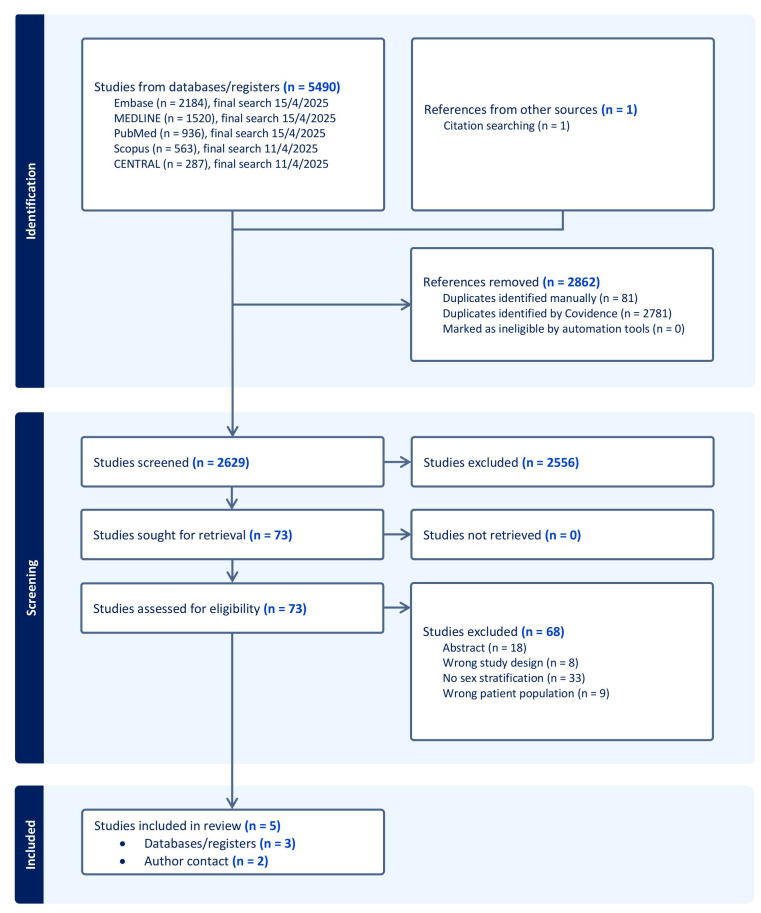
PRISMA flow-chart for the study selection process.

The search process is visualized in the PRISMA Flowchart (Figure 2). The primary search yielded 5490 studies. After the removal of duplicates, 2629 studies remained and were screened for titles and abstracts. A total of 73 full-text manuscripts were assessed and compared to the eligibility criteria of this study. Of these, only three studies were included [22,23,24]. Following this, corresponding authors of 11 eligible studies were contacted, resulting in the additional inclusion of the two datasets by Fleury et al. [20] and Viitala et al. [21]. An overview of the studies included is provided in Table 1. A complete list of studies excluded after full-text review, including reasons for exclusion, is provided in the Supplementary Materials Section S1, Table S1.4.1.

With the exception of Obel et al. [24], that relied on prospectively collected data in a population-based setting, all other studies were historical cohort studies with study populations consisting of patients.

Across the included studies, imaging practices varied substantially. Two of the five studies used CT as the sole imaging modality. CT was used to some extent in all studies except in Fleury et al. [20] where aortic growth rate was evaluated with only TTE. Cheung et al. [22] was the only study to use MRI, and it was reported as being used for 10% of the whole cohort at baseline. In addition to CT and MRI, Cheung et al. [22] used echocardiography without specifying whether transthoracic or transesophageal echocardiography was used. Regarding technical standards, ECG-gated CT was used in studies by Kauhanen et al. [23] and Obel et al. [24] whereas Viitala et al. [21] and Cheung et al. [22] stated adherence to guidelines that do not explicitly require ECG-gating, leaving its use uncertain. Among the included studies, Kauhanen et al. [23] was the only one to use CT angiography.

All studies reported that aortic measurements were performed by trained personnel, but they differed in how thoroughly measurement validation procedures were described. Some studies reported inter- or intra-observer verification, whereas others only stated that trained staff performed the assessments without additional detail.

### 3.2. Baseline Characteristics

Participant characteristics from the included studies are summarized in Table 2. Definitions of baseline characteristics varied between the studies. All studies reported the age, height, weight, body mass index, and body surface area of the participants. However, Kauhanen et al. [23] reported these only for the total cohort, whereas Obel et al. [24] provided data separately for females and males, but not for the total cohort. The median ages across all studies were comparable. Kauhanen et al. [23] did not report sex-stratified age. Among the remaining studies that reported sex-stratified mean ages, Viitala et al. [21] and Cheung et al. [22] presented female cohorts with a higher mean age than in males. In Cheung et al. [22] the female cohort had a notably higher median age than the male cohort. All studies reported the smoking status of the participants, but classification and reporting varied. Fleury et al. [20], Viitala et al. [21] and Cheung et al. [22] combined former and current smokers, whereas Obel et al. [24] divided participants into current, former, and non-smokers.

In terms of mean baseline diameters, all studies except Kauhanen et al. [23] reported sex-stratified baseline measurements. Only Cheung et al. [22] combined measurements of the aortic root with those of the ascending aorta. Obel et al. [24] was the only study to categorize patients into size groups based on the absolute diameter of the aorta.

### 3.3. Follow-Up Time and Data Collection Time Frame

Cheung et al. [22] included participants, with minimum two measurements at least six months apart. The length of the study period was two years, from February 2014 to February 2016. Follow-up times were reported separately for females and males. The mean follow-up time was 3.1 ± 2.8 years, which was similar between men and women (*p* = 0.61). Kauhanen et al. [23] had a mean imaging follow-up time of 3.4 ± 1.6 years, participants were included between January 2013 and December 2018. The study by Obel et al. [24] was based on data from two population-based, multicenter, randomized screening trials, DANCAVAS I and II including participants from 2014 to 2018. The median-follow up time was 4.5 years [Q1–Q3: 3.4–4.7 years]. Fleury et al. [20] analyzed data from patients with at least mild aortic stenosis enrolled in the prospective observational Metabolic Determinants of the Progression of Aortic Stenosis (PROGRESSA) study, conducted between 2005 and 2022. Participants were followed for up to five years. The exclusion criteria applied by Fleury et al. [20] were generally aligned with those of the present review; however, their published reporting did not provide a numeric sex-stratified growth rate for the subgroup of asymptomatic patients with a tricuspid aortic valve (TAV). Consequently, a dataset provided directly by Fleury et al. [20], in which patients with bicuspid aortic valves had been excluded, was analyzed by the authors of this article to retrieve sex-stratified growth rates. Viitala et al. [21] identified all patients with aTAA who had been followed at the Central Hospital of North Karelia in Eastern Finland between June 2007 and July 2023. Patients included had undergone at least two chest CT scans or two TTE examinations, with a minimum interval of 12 months between imaging studies. As Viitala et al. [21] did not report sex-stratified growth rate estimates for non-syndromic patients with tricuspid aortic valves, the corresponding author provided a dataset restricted to measurements from the tubular ascending aorta. As with the dataset from Fleury et al. [20], growth rates were analyzed by the authors of the present review.

### 3.4. Absolute Yearly Growth Rates

Three of the five included studies concluded a statistically significant difference in aTAA growth rates between females and males. Results from the studies included are presented in Table 3.

Cheung et al., Obel et al. and the results calculated from the dataset provided by Viitala et al. [21,22,24] showed that female sex was associated with higher growth rates of aTAA, but only the two first reported were significant, with *p*-values of 0.0009 and 0.012 whereas the *p*-value for Viitala et al. [21] was non-significant. Growth rate according to Cheung et al. [22] was 1.74 ± 1.17 mm/year for females and 0.55 ± 0.59 mm/year for males. Obel et al. [24] found that the growth rate for females was 0.13 ± 0.3 mm/year and for males 0.07 ± 0.5 mm/year. In contrast, Fleury et al. [20] and Kauhanen et al. [23] both found higher growth rates in males, although only the latter study found this difference to be statistically significant. Kauhanen et al. [23] reported growth rates for the mid-ascending aorta being 0.2 ± 0.5 mm/year in males and −0.7 ± 0.4 mm/year for females. Kauhanen et al. [23] attribute the negative growth rate to measurement variability exceeding the average slow rate of biological growth.

**Figure 3:**
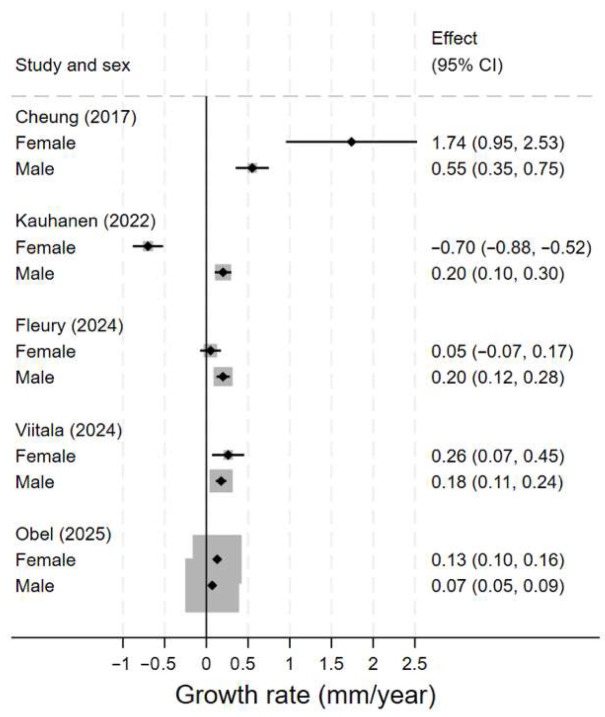
Forest plot for growth rate of ascending aortic aneurysm stratified by sex [20,21,22,23,24].

The results varied considerably between studies (Figure 3). Most notably, Cheung et al. [22] reported a much higher growth rate for females (1.74 ± 1.17 mm/year) compared with males (0.55 ± 0.59 mm/year), and this value was also markedly higher than the growth rates reported in any of the other studies.

### 3.5. Results of the QoS Evaluation

The main findings of the QoS evaluation are presented in Table 4. None of the studies scored full points. The scores reflect the degree of compliance with standardized definitions and reporting practices in aortic aneurysm imaging studies.

Obel et al., Kauhanen et al. and Viitala et al. [21,23,24] were rated as good quality studies, whereas scores for Fleury et al. and Cheung et al. [20,22] correspond to moderate quality. Cheung et al. [22] showed the lowest score of 3.5 with most points lost on items evaluating risk of bias (RoB). When looking at the items addressing RoB (items 5,6,7), none of the three included studies scored full points on all three items.

The most consistently met criteria across studies were item 3, concerning the description of measurement techniques and item 7, looking at the adequate reporting of statistical significance. In addition, all studies reported on their funding sources and declared conflicts of interest. None of the included studies scored on item 4 by ensuring more than two scans for all participants.

## 4. Discussion

The findings of this systematic review support sex-related differences in growth rates of aTAAs in non-syndromic individuals with normal aortic valve morphology. However, to reach an evidence-based consensus, future research needs to be more standardized. Importantly, the included studies were not methodologically superimposable. Differences in imaging modality, ECG-gating, measurement location along the ascending aorta, cohort composition, and follow-up duration precluded the meaningful pooling of growth estimates. Consequently, no meta-analysis was performed, and growth rates were interpreted within their individual methodological context to avoid false precision. This is in line with a previous systematic review from 2016 by Oladokun et al. [25], that called for more standardized methods when assessing the growth of TAAs. Later, in 2024, Cozijnsen et al. [5] concluded that studies published before 2016 were less standardized than those after. Importantly, they found that more recent studies reported slower growth rates than those of older landmark studies. This finding reflects what is seen when reviewing the study by Cheung et al. [22] from 2017, which had the lowest QoS score, corresponding to low adherence to standardization and a risk of bias while simultaneously reporting the highest growth rates among all the studies included.

To the best of our knowledge, previous reviews have not reported sex-specific growth rates of aTAAs specifically for non-syndromic patients without bicuspid aortic valves. While some studies present sex-stratified data, these often include patients with bicuspid aortic valves, making it difficult to isolate normative growth rates.

The most recent systematic review on thoracic aortic aneurysm growth, published in 2025 by Henry et al. [13], included an analysis of growth rates for non-syndromic aTAAs, with the group defined as “sporadic” being the most comparable to the population considered in this review. In their synthesis, studies were grouped as sporadic when the majority of participants met the criteria, even if some included individuals with BAV or prior aortic surgery and valve replacement. The pooled mean growth rate for sporadic ascending aneurysms was 0.46 mm/year (95% CI, 0.24–0.68), closely aligning with the findings of Viitala et al. [21] (0.31 mm per year). Cheung et al. [22] reported a markedly higher growth rate in females (1.74 ± 1.17 mm/year), while their reported growth rate for males (0.55 ± 0.59 mm/year) was more consistent with the other studies. Henry et al. found only five studies reporting growth rates of similar rates (>1 mm/year). Among the most commonly reported predictors of aortic growth in sporadic aTAA, Henry et al. [13] noted that 13 studies evaluated sex differences, of these eight found no association, four identified significantly higher growth rates in females, and one study found higher rates in males, limited to the aortic root rather than the mid-ascending segment, reflecting heterogeneity in reporting.

Factors contributing to the considerable differences in results across the studies included are likely due to the variation in how adherent the studies were to the use of homogeneous techniques and measurements protocols, including variation in minimum follow-up time. The greatest variation in growth rate was reported in the study by Cheung et al. [22], which also was the study least adherent to standardized methods. A potentially contributing factor to the faster growth rate reported by Cheung et al. [22] could be that their patient population presents the largest baseline diameters compared to the patient populations of the other studies. Important to note is that Cheung et al. [22] reported measurements from the root of the aorta together with those of the ascending aorta, and although they found 71% of the largest dilatations at the level of the ascending aorta and 29% at the root, this may be an underlying reason why their measured growth rate appears as an outlier. It is also notable that this study is the oldest of those included.

According to the 2024 ESC guidelines [9], CT scans are highly recommended when evaluating TAAs. As the guidelines recommend measuring diameter at end-systole, the CT should be ECG-gated. Lack of ECG-gating can lead to measurement errors, as the end-systolic diameters have been shown to be significantly higher than the end-diastolic measurements [26]. The use of non-concordant imaging modalities contributes to measurement variability [27] and when combined with a small study population, as in Cheung et al. [22], there is an increased probability of measurement errors being amplified. CT was the most commonly used modality (in 57%) others were echocardiography and MRI. CT was not reported to have been ECG-gated.

Differences in imaging modality, ECG-gating, and measurement protocols represent important sources of measurement variability in studies assessing ascending aortic growth. [28,29] In the present review, substantial heterogeneity was observed across studies with respect to imaging techniques and technical standards, which may influence absolute growth estimates independently of biological factors. These methodological aspects were explicitly accounted for in the quality assessment and constitute an important non-biological source of inter-study heterogeneity. Importantly, no pooled meta-analyses were performed, and growth rates were therefore not averaged across heterogeneous measurement methods. Instead, differences were interpreted within their methodological context rather than as precise quantitative contrasts. This conservative approach was chosen to avoid false precision and to ensure that reported sex-specific growth rates were interpreted within the context of methodological as well as biological and clinical heterogeneity.

Beyond methodological considerations, aTAA development is increasingly recognized as a biologically active process involving chronic inflammation, immune activation, and extracellular matrix remodeling. Inflammatory cell infiltration and matrix degradation have been demonstrated in aneurysmal aortic tissue and may influence disease progression and growth behavior [15]. Moreover, immune and inflammatory responses are known to differ between females and males across cardiovascular diseases, potentially influenced by differences in sex-specific hormones, genetic factors and gender-related risk behavior [30,31]. Such differences could plausibly contribute to inter-individual variability in aortic disease progression, including observed differences in growth patterns. Although immune or inflammatory biomarkers were not assessed in the present review, these mechanisms likely provide relevant biological context for the observed heterogeneity in growth rates between studies and between sexes.

Previous literature has identified age as a factor influencing the growth of the thoracic aorta, and this highlights the importance of reporting age stratified by sex [32]. For example, Kauhanen et al. [23] did not report age for female and male cohorts and given that their population comprises mostly males (80.4%), it was not possible to assess the influence of age on the sex differences. Cheung et al. [22] only included patients in their cohort and reported a higher mean age for females. This might reflect the clinical trend of females presenting with aTAA at an older age, as seen in previous studies [33].

Several clinical and hemodynamic factors have been proposed as potential modifiers of thoracic aortic disease progression, including blood pressure control, heart rate and rate-controlling therapy, lipid profile, and inflammatory or atherosclerotic vascular burden [25,34]. However, for growth of the ascending thoracic aorta, evidence supporting consistent associations between these factors and diameter progression remains limited and are often inconsistent across studies [35].

Multiple studies included in the present review evaluated traditional cardiovascular risk factors—such as hypertension, smoking, and diabetes—without demonstrating significant associations with growth rate. Moreover, optimal blood pressure targets and pharmacological strategies for patients with ascending thoracic aortic aneurysms are not well established, and recent data have questioned the protective role of beta-blocker therapy in this population [36,37]. Conversely, diabetes mellitus has been associated with a lower risk of thoracic aortic dissection, underscoring that classical cardiovascular risk factors do not uniformly translate into aortic growth dynamics [4].

Aortic valve morphology represents a well-established determinant of ascending aortic growth [35]; however, this factor was intentionally controlled for by restricting inclusion to patients with tricuspid aortic valves, thereby minimizing confounding by design.

Indexed growth rates play a role in outcomes, as seen in the DisSEXion study, which compared indexed and non-indexed growth rates for the sinuses of Valsalva and the tubular ascending aorta [6]. When growth was indexed for height using the Aortic Height Index, the rates were comparable between sexes, whereas the absolute growth rate was higher in males. This systematic review reports absolute growth rates, since indexed growth rates were only used by Cheung et al. [22]. Assuming a normal distribution of height in all cohorts, females tend to be shorter than males. Without indexing, there is a risk that the relative progression in females may be underestimated.

Importantly, absolute ascending aortic growth rate could be interpreted as a surrogate measure rather than a direct marker of clinical risk. While growth rate is widely used in surveillance and decision-making, adverse aortic events such as dissection or rupture are not determined by diameter progression alone [9]. Instead, these outcomes reflect a complex interaction between aortic wall biology, hemodynamic load, medical therapy, and patient-specific vulnerability. Consequently, sex-specific differences in absolute growth rates, when observed, should not be interpreted as evidence of intrinsic sex-related differences in aneurysm behavior in isolation, but rather as the net result of biological, therapeutic, and methodological factors operating within the constraints of the available data. 

The lack of reporting on sex-stratified data is the main reason for the small number of included articles in this review. As seen in the PRISMA diagram (Figure 2), the most common exclusion criteria were the absence of sex-stratified data. Several otherwise eligible studies contained potentially valuable information on sex-specific aTAA growth but did not report the data in a disaggregated form [6,20,38,39,40,41,42,43]. It is plausible that subgroup data, including sex-stratification, could have been extracted by the authors, but this was not reported in the published articles. After contacting the corresponding authors of articles where we deemed it plausible for further analysis of already obtained data, we succeeded in establishing a collaboration with Fleury et al. and Viitala et al. [20,21] resulting in the addition of two sets of results.

A notable limitation of this review is the absence of a meta-analysis, primarily due to the restricted number of eligible studies and the substantial heterogeneity among those included. Further limitations include the sparse and inconsistent reporting of clinical endpoints, such as aortic dissection and surgical intervention, across the included studies, with stratification by sex rarely performed. Additionally, the majority of studies were not specifically designed to assess clinical outcomes, thereby constraining the ability to correlate observed aneurysm growth rates with adverse aortic events. As a result, this review focuses on absolute aneurysm growth as the primary outcome, and the findings should not be interpreted as directly indicative of sex-specific differences in clinical risk.

Regarding the relatively narrow and contemporary publication time frame (2017–2025) of the included studies, it could be argued that this reduces the likelihood of temporal variations in imaging technology or clinical practice substantially influencing the observed differences in growth rates.

In summary, findings from this systematic review align with earlier studies, including recent work by Samman et al. [31] and the AHA Scientific Statement [30] which both call for more high-quality studies focusing on female cardiovascular health. Accordingly, the findings of this review should be interpreted as descriptive, reflecting both biological variability and methodological heterogeneity rather than definitive sex-specific differences in aneurysm growth. Future studies should apply standardized, sex-stratified methodologies and longer follow-up periods to better define normative aTAA growth that guides clinical decision-making.

## 5. Conclusions

This systematic review assessed the sex-specific growth rates of ascending thoracic aortic aneurysms (aTAAs) in non-syndromic adult patients without prior aortic surgery or valve replacement. Three of the five included studies reporting higher growth rates in females. Of these three, only one study was rated as good quality and reported a statistically significant difference (*p* < 0.05). Of the two studies reporting higher growth rates in males, only one was of good quality and reported a statistically significant difference. The incoherent findings, together with differences in imaging protocols and techniques, cohort definitions, and outcome reporting, limit the strength of the conclusions; thus, the overall evidence remains inconsistent.

## 6. Future Directions

Future studies should apply standardized, sex-stratified methodologies and longer follow-up periods, preferably exceeding one year, to better define normative aTAA growth that guides clinical decision-making. In addition, future studies should integrate standardized reporting of clinical, hemodynamic, and therapeutic variables to enable a more comprehensive evaluation of factors influencing ascending aortic growth.

## Figures and Tables

**Table 1:** Studies included in this systematic review.

1st Author Country, Pub. Year	Study Design	Study Population	Imaging Modality Used
Cheung, Canada, 2017 [22]	Historical patient cohort	Patients	Echocardiography ^a^, CT, MRI
Kauhanen, Finland, 2022 [23]	Historical patient cohort	Patients	CTA
Fleury, Canada, 2024 [20]	Prospective cohort	Patients	TTE
Viitala, Finland, 2024 [21]	Historical patient cohort	Patients	CT, TTE
Obel, Denmark, 2025 [24]	Prospective cohort	Population and patients mixed	CT

Footnotes: CT = Computed Tomography, CTA = Computed Tomography Angiography, MRI = Magnetic Resonance Imaging, TTE = Trans Thoracic Echocardiography. ^a^ Type of echocardiography (transthoracic or transesophageal) not specified in the original study.

**Table 2:** Common baseline characteristics of the participants in the included studies.

Study	Cohort	*n*	Age (Years)	BSA (m^2^)	Baseline (mm)	Smoking, *n* (%)			HTN, *n* (%)	DM, *n* (%)
					asc . aorta	Current	Former	Never		
Cheung et al. ^a^ [22]	Total	82	63.8 ± 11.8	2.0 ± 0.2	45.6 ± 4.3 ^b^	48 (58.5) ^c^	NR	NR	40 (48.8)	4 (4.9)
	Female	21	67.7 ± 10.7	1.9 ± 0.2	45.1 ± 5.1 ^b^	14 (66.7) ^c^	NR	NR	9 (42.9)	1 (4.8)
	Male	61	62.4 ± 11.9	2.1 ± 0.2	45.8 ± 4.0 ^b^	34 (55.7) ^c^	NR	NR	31 (50.8)	3 (4.9)
Kauhanen et al. ^a^ [23]	Total	143	64.0 ± 10.0	2.0 ± 0.2	43.7 ± 4.1	16 (11.2)	NR	NR	96 (67.1)	18 (12.6)
	Female	28	NR	NR	NR	NR	NR	NR	NR	NR
	Male	115	NR	NR	NR	NR	NR	NR	NR	NR
Fleury et al. ^d^ [20]	Total	222	70.0 ± 8.0	1.9 ± 0.2	34.5 ± 4.3	NR	NR	NR	196 (88.3)	68 (30.6)
	Female	52	69.4 ± 8.7	1.7 ± 0.1	32.3 ± 3.9	NR	NR	NR	43 (83.0)	13 (25.0)
	Male	170	70.2 ± 7.8	1.9 ± 0.2	35.2 ± 4.2	NR	NR	NR	153 (90.0)	55 (32.4)
Viitala et al. ^d^ [21]	Total	148	64.9 ± 9.8	2.04 ± 0.3	44.0 ± 4.4	77 (36.8) ^a^	NR	NR	128 (86.5)	26 (17.6)
	Female	29	67.9 ± 9.1	1.79 ± 0.2	44.7 ± 3.3	NR	NR	NR	27 (93.1)	3 (10.3)
	Male	119	64.2 ± 9.8	2.09 ± 0.2	43.8 ± 4.6	NR	NR	NR	101 (84.9)	23 (19.3)
Obel et al. [24]	Total	2026	NR	NR	38.3 ± 5.3	NR	NR	NR	NR	NR
	Female	460	68.5 ± 3.2	1.8 ± 0.2	33.9 ± 3.5	47 (10.3)	162 (35.4)	249 (54.4)	247 (53.7)	30 (6.5)
	Male	1566	69.4 ± 3.1	2.09 ± 0.2	39.6 ± 5.0	263 (16.8)	857 (54.8)	444 (28.4)	920 (58.8)	222 (14.2)

All measurements are given as means with standard deviation unless stated otherwise. ^a^ Values specific to the analyzed sub-population were not reported. Values refer to the entire cohort. ^b^ Measurements from root of the aorta reported together with those of ascending aorta. ^c^ Current and former smokers grouped together by defining smoking as having smoked over 100 cigarettes in the past. ^d^ Values calculated by authors of current article from data acquired from Fleury et al. and Viitala et al. DM: Diabetes Mellitus. HTN: Hypertensio Arterialis. NR: Not Reported.

**Table 3:** Results from the included studies—annual growth rates for ascending aortic aneurysms compared between females and males.

Study	Cohort	Sub-Group Size, *n*	Absolute Growth Rate (mm/Year)	Follow-Up Time	Result
Cheung et al. [22]	Total	47	0.74 ± 0.85	3.1 ± 2.8	Female gender associated with increased growth rate, *p* = 0.0009
	Female	11	1.74 ± 1.17	3.7 ± 3.5	
	Male	36	0.55 ± 0.59	2.9 ± 2.5	
Kauhanen et al. [23]	Total	121	0.2 ± 0.5	3.4 ± 1.6 ^a^	Male gender associated with increased growth rate, *p* = 0.047
	Female	21	−0.7 ± 0.4	NR	
	Male	100	0.2 ± 0.5	NR	
Fleury et al. ^b^ [20]	Total	222	0.22 ± 0.59	3.5 ± 1.5	Difference in growth rates not statistically significant, *p* = 0.056
	Female	52	0.07 ± 0.55	3.6 ± 1.5	
	Male	170	0.27 ± 0.60	3.5 ± 1.5	
Viitala et al. ^b^ [21]	Total	148	0.31 ± 0.80	4.8 ± 3.5	Difference in growth rates not statistically significant, *p* = 0.23
	Female	29	0.44 ± 0.67	4.3 ± 2.6	
	Male	119	0.27 ± 0.83	5.0 ± 3.7	
Obel et al. [24]	Total	2026	0.08 ± 0.5	4.5 ^c^	Female gender associated with increased growth rate, *p* = 0.012
	Female	460	0.13 ± 0.3	NR	
	Male	1566	0.07 ± 0.5	NR	

Growth rates are presented as mm per year along with the corresponding standard deviations. ^a^ mean follow-up time for imaging. ^b^ values calculated by the authors of this article from data received from Fleury et al. and Viitala et al. Wilcoxon test for *p*-value. ^c^ reported as median follow-up time, [Q1–Q3: 3.4–4.7 years]. NR: Not Reported.

**Table 4:** Quality of the included studies (QoS).

Study	Aneurysm Definition	Imaging Method	Measurement Technique	>2 Time Points	Follow-Up	Missing Data	Statistical Analysis	Points Out of 7
Items 1–7	1.	2.	3.	4.	5.	6.	7.	
Cheung et al., 2017 [22]	1	0.5	0.5	0	0	0	1	3.0
Kauhanen et al., 2022 [23]	0	1	1	0	1	0.5	1	4.5
Fleury et al., 2024 [20]	0	1	1	0	0	0	1	3.0
Viitala et al., 2024 [21]	0	1	1	0	1	1	1	5.0
Obel et al., 2025 [24]	1	1	1	0	1	1	1	6.0

Number with corresponding item: 1. Is the definition of the ascending thoracic aortic aneurysm based on a personalized, indexed aortic diameter? 2. Was the imaging method standardized and clearly described? 3. Is the measurement technique described? 4. Were there >2 measurement time points? 5. Was the follow-up period sufficient, ≥1 year? (Re-adjusted from MINORS, item 6). 6. Was loss during follow-up clearly described with <5% unexplained missing data? (Re-adjusted from MINORS, item 7). 7. Were confidence intervals or statistical significance reported in the original article? (Re-adjusted from MINORS, item 8). Items 4 and 5 give either 0 or 1 point, whilst other items also yield half-points. See Supplementary Materials for more details.

## Data Availability

This study is a systematic review. All data underlying the three published studies included in the review are publicly available from the original publications. Two additional datasets (Fleury et al. and Viitala et al. [20,21]) were obtained directly from the corresponding authors of those studies and are not publicly shareable due to data-protection restrictions; these datasets may be requested from the original investigators. No new data was generated by the authors of this article.

## Supplementary Materials

The following supporting information can be downloaded at: https://www.mdpi.com/article/10.3390/diagnostics16060916/s1, Supplementary Materials Sections S1–S3. S1: Search Strategy and Study Selection; S2: Quality and Risk of Bias Evaluation of Included Studies; S3: Completed PRISMA 2020 Guideline Checklists.

## Abbreviations

The following abbreviations are used in this manuscript:

AA	Ascending Aorta
AHA	American Heart Association
aTAA	Ascending Thoracic Aortic Aneurysm
BSA	Body Surface Area
CT	Computed Tomography
ECG	Electrocardiogram
ESC	European Society of Cardiology
MRI	Magnetic Resonance Imaging
RoB	Risk Of Bias
TAA	Thoracic Aortic Aneurysm
