# Supplementary material for: Sex-Specific Growth Rates of Ascending Thoracic Aortic Aneurysms in Non-Syndromic Patients: A Systematic Review
Source: Diagnostics (Basel). 2026 Mar 19;16(6):916. doi: 10.3390/diagnostics16060916 (PMC13024991; doi:10.3390/diagnostics16060916)
Supplement: Supplementary file 1 [file diagnostics-16-00916-s001.zip › diagnostics-4179266-supplementary.pdf]

Supplementary Material Index

S1 SEARCH STRATEGY AND STUDY SELECTION 1
S1.1 Search Strategy 1
S1.2 Dilatation/Dilation 1
S1.3 Search Strings 1
S1.4 Comprehensive List of Excluded Studies 11
S2 QUALITY AND RISK OF BIAS EVALUATION OF INCLUDED STUDIES 17
S2.1 Description of the Quality of Studies Evaluation (QoS) Tool 17
S2.2 Definition of Each Item and Scoring Rationale 17
S3 COMPLETED PRISMA 2020 GUIDELINE CHECKLISTS 19
S3.1 PRISMA 2020 ABSTRACTS CHECKLIST 19
S3.2 PRISMA COMPREHENSIVE CHECKLIST 20
REFERENCE LIST FOR SUPPLEMENTARY MATERIALS 21

S1 Search Strategy and Study Selection

S1.1 Search Strategy

The searches were conducted in the following five databases Ovid EMBASE, Ovid MEDLINE, PubMed, Elsevier Scopus and Cochrane Central Register of Controlled Trials. Search strings for each database is listed below (S1.3) with date and time for conducted search in addition to the number of results yielded.

S1.2 Dilatation/Dilation

Truncation was used in the search string when searching for the term *dilatation,* as the terms *dilatation* and dilation are frequently - albeit incorrectly - used interchangeably in the research literature [1]. Although the correct medical term in the context of aneurysms would be dilatation [2], the inaccurate term dilation appears frequently in the same context. To ensure retrieval of relevant studies, the truncation accounted for both variants.

The above-mentioned terms were included in the search strategy since the definition of aneurysm varies across the literature and also to ensure capture of studies identifying aneurysms on the basis of non-absolute, indexed aortic diameters.

S1.3 Search Strings

CENTRAL
11/4/2025 - kl 13:00
Yielded 287 results
https://www.cochranelibrary.com/advanced-search/search-manager?search=7676895

View fewer lines | Print search history

#1	MeSH descriptor: [Aorta, Thoracic] explode all trees	MeSH	229
#2	MeSH descriptor: [Dissection, Thoracic Aorta] explode all trees	MeSH	3
#3	MeSH descriptor: [Aortic Aneurysm, Thoracic] explode all trees	MeSH	158
#4	MeSH descriptor: [Aneurysm, Ruptured] explode all trees	MeSH	313
#5	(ascending NEAR/2 aort*):ti,ab	Limits	529
#6	(thoracic aort*):ti,ab	Limits	885
#7	(TAA):ti,ab	Limits	290
#8	(aTAA):ti,ab	Limits	9
#9	(thoracic aort* aneurysm*):ti,ab	Limits	193
#10	(ascending aort* aneurysm*):ti,ab	Limits	65
#11	(aort* NEAR/2 dissection*):ti,ab	Limits	489
#12	(aort* NEAR/2 aneurysm*):ti,ab	Limits	1760
#13	(dissect* NEAR/2 aneurysm*):ti,ab	Limits	99
#14	(aort* root):ti,ab	Limits	471
#15	#1 OR #2 OR #3 OR #4 OR #5 OR #6 OR #7 OR #8 OR #9 OR #10 OR #11 OR #12 OR #13 OR #14	Limits	4200
#16	(growth rate*):ti,ab	Limits	13501
#17	(dilat* rate*):ti,ab	Limits	5191
#18	(progression rate*):ti,ab	Limits	40230
#19	(expansion rate*):ti,ab	Limits	2679
#20	(millimeter progression):ti,ab	Limits	53
#21	(diameter progression):ti,ab	Limits	1470
#22	(natural history and aneurysm*):ti,ab	Limits	18
#23	#16 OR #17 OR #18 OR #19 OR #20 OR #21 OR #22	Limits	58095
#24	#15 AND #23	Limits	287

✖ Clear all

Highlight orphan lines

SCOPUS
11/4/2025 - kl:11:15
Yielded 563 results
(TITLE-ABS ("acute aort* syndrome" OR "dissecting aort* aneurysm" OR "thoracic aort* dissection*" OR "type A aort* dissection" OR "thoracic aort*" OR ("ascending W/2 aort*") OR taa OR ataa OR "thoracic aort* aneurysm*" OR "ascending aort* aneurysm" * OR ("aort* W/2 dissection*") OR ("aort* W/2 aneurysm*") OR ("dissect* W/2 aneurysm*") OR "aort* root")) AND (TITLE-ABS ("growth rate*" OR "dilat* rate*" OR "progression rate*" OR "expansion rate*" OR "millimeter progression" OR "diameter progression" OR ("natural history" AND aneurysm*)))

PUBMED
15/4/25 - kl 12:15
yielded 936 results
("Aorta, Thoracic"[MeSH Terms]
OR "Aortic Aneurysm, Thoracic"[MeSH Terms]
OR Aneurysm, Thoracic Aortic[MeSH Terms] OR aneurysm, ruptured aortic[MeSH Terms]
OR "Thoracic Aortic"[Title/Abstract:~2]

OR "Thoracic Aorta"[Title/Abstract:~2]
OR TAA[Title/Abstract]
OR aTAA[Title/Abstract]
OR "Thoracic Aortic Aneurysm"[Title/Abstract]
OR "Thoracic Aortic Aneurysms"[Title/Abstract]
OR "Dissect* Aneurysm*"[Title/Abstract]
OR "Dissecting Aneurysm"[Title/Abstract]
OR "Dissecting Aneurysms"[Title/Abstract]
OR "Aort* Dissect*"[Title/Abstract]
OR "Aortic Dissection"[Title/Abstract]
OR "Aortic Dissections"[Title/Abstract]
OR "Aortic Root"[Title/Abstract]
OR "Aortic Rupture"[Title/Abstract]
OR "Ascending Aortic"[Title/Abstract:~2]
OR "Ascending Aorta"[Title/Abstract:~2]
OR thoracic* aort*[Title/Abstract])
AND
(Growth rate*[Title/Abstract]
OR dilat* rate*[Title/Abstract]
OR progression rate[Title/Abstract]
OR (natural history[Title/Abstract] AND aneurysm*[Title/Abstract])
OR diameter progression[Title/Abstract]
OR millimeter progression[Title/Abstract]
OR expansion* rate*[Title/Abstract])

OVID MEDLINE(R)
15/4/2025 - kl.12:00
Yielded 1520 results
https://proxy1-bib.sdu.dk:2048/login?url=https://ovidsp.ovid.com/ovidweb.cgi?T=JS&NEWS=N&PAGE=main&SHAREDSEARCHID=3UwP9BR3rFsgWW66y2hWMMCsDrp0FcCQfcN5pGhlF7DrofUIvQICgqVi2d894fjUK

#	Query	Results from 15 Apr 2025
1	thoracic aorta/	38,852
2	thoracic aorta aneurysm/	16,485
3	aortic dissection/	22,508
4	thoracic aortic wall/	0

5	"aortic root aneurysm"/	13
6	ascending aortic aneurysm/	42
7	acute aortic syndrome/	53
8	dissecting aortic aneurysm/	0
9	thoracic aortic dissection/	39
10	type a aortic dissection/	0
11	thoracic aort*.ti,ab.	24,395
12	(ascending adj3 aort*).ti,ab.	20,531
13	TAA.ti,ab.	7,642
14	aTAA.ti,ab.	227
15	thoracic aort* aneurysm*.ti,ab.	4,530
16	ascending aort* aneurysm*.ti,ab.	1,588
17	(aort* adj3 dissection*).ti,ab.	22,456
18	(aort* adj3 aneurysm*).ti,ab.	45,988
19	(dissect* adj3 aneurysm*).ti,ab.	8,789
20	aort* root.ti,ab.	12,407

21	1 or 2 or 3 or 4 or 5 or 6 or 7 or 8 or 9 or 10 or 11 or 12 or 13 or 14 or 15 or 16 or 17 or 18 or 19 or 20	138,804
22	growth rate/	0
23	growth rate*.ti,ab.	86,781
24	dilat* rate*.ti,ab.	325
25	progression rate*.ti,ab.	5,685
26	expansion rate*.ti,ab.	1,599
27	millimeter progression.ti,ab.	1
28	diameter progression.ti,ab.	36
29	(natural history and aneurysm*).ti,ab.	1,736
30	22 or 23 or 24 or 25 or 26 or 27 or 28 or 29	95,880
31	21 and 30	1,513
32	thoracic aorta/	38,852
33	thoracic aorta aneurysm/	16,485
34	aortic dissection/	22,508
35	"aortic root aneurysm"/	13
36	acute aortic syndrome/	53

37	dissection, thoracic aorta/	39
38	dissection, ascending aorta/	19
39	aortic aneurysm, thoracic/	16,485
40	aneurysm, ascending aorta/	42
41	aortic rupture/	10,488
42	thoracic aort*.ti,ab.	24,395
43	(ascending adj3 aort*).ti,ab.	20,531
44	TAA.ti,ab.	7,642
45	aTAA.ti,ab.	227
46	thoracic aort* aneurysm*.ti,ab.	4,530
47	ascending aort* aneurysm*.ti,ab.	1,588
48	(aort* adj3 dissection*).ti,ab.	22,456
49	(aort* adj3 aneurysm*).ti,ab.	45,988
50	(dissect* adj3 aneurysm*).ti,ab.	8,789
51	aort* root.ti,ab.	12,407
52	growth rate*.ti,ab.	86,781
53	dilat* rate*.ti,ab.	325

54	progression rate*.ti,ab.	5,685
55	expansion rate*.ti,ab.	1,599
56	millimeter progression.ti,ab.	1
57	diameter progression.ti,ab.	36
58	(natural history and aneurysm*).ti,ab.	1,736
59	32 or 33 or 34 or 35 or 36 or 37 or 38 or 39 or 40 or 41 or 42 or 43 or 44 or 45 or 46 or 47 or 48 or 49 or 50 or 51	141,296
60	52 or 53 or 54 or 55 or 56 or 57 or 58	95,880
61	59 and 60	1,520
62	thoracic aorta/	38,852
63	thoracic aorta aneurysm/	16,485
64	aortic dissection/	22,508
65	"aortic root aneurysm"/	13
66	acute aortic syndrome/	53
67	dissection, thoracic aorta/	39
68	dissection, ascending aorta/	19
69	aortic aneurysm, thoracic/	16,485

70	aneurysm, ascending aorta/	42
71	aortic rupture/	10,488
72	thoracic aort*.ti,ab.	24,395
73	(ascending adj3 aort*).ti,ab.	20,531
74	TAA.ti,ab.	7,642
75	aTAA.ti,ab.	227
76	thoracic aort* aneurysm*.ti,ab.	4,530
77	ascending aort* aneurysm*.ti,ab.	1,588
78	(aort* adj3 dissection*).ti,ab.	22,456
79	(aort* adj3 aneurysm*).ti,ab.	45,988
80	(dissect* adj3 aneurysm*).ti,ab.	8,789
81	aort* root.ti,ab.	12,407
82	growth rate*.ti,ab.	86,781
83	dilat* rate*.ti,ab.	325
84	progression rate*.ti,ab.	5,685
85	expansion rate*.ti,ab.	1,599
86	millimeter progression.ti,ab.	1

87	diameter progression.ti,ab.	36
88	(natural history and aneurysm*).ti,ab.	1,736
89	62 or 63 or 64 or 65 or 66 or 67 or 68 or 69 or 70 or 71 or 72 or 73 or 74 or 75 or 76 or 77 or 78 or 79 or 80 or 81	141,296
90	82 or 83 or 84 or 85 or 86 or 87 or 88	95,880
91	89 and 90	1,520

OVID EMBASE
15/4/2025 - kl.12:10
Yielded 2,184 hits
https://proxy1-bib.sdu.dk:/login?url=https://ovidsp.ovid.com/ovidweb.cgi?T=JS&NEWS=N&PAGE=main&SHAREDSEARCHID=5AmWpr0MxVPQ2BMwxG1dauHUROcV6tU0J0F7pZEqhi5xSerDdI6WqfkPKbKutelRV

#	Query	Results from 15 Apr 2025
1	thoracic aorta/	28,607
2	thoracic aorta aneurysm/	10,866
3	aortic dissection/	28,843
4	thoracic aortic wall/	76
5	"aortic root aneurysm"/	297
6	ascending aortic aneurysm/	1,669

7	acute aortic syndrome/	1,390
8	dissecting aortic aneurysm/	2,139
9	thoracic aortic dissection/	396
10	type a aortic dissection/	1,984
11	thoracic aort*.ti,ab.	34,256
12	(ascending adj3 aort*).ti,ab.	31,447
13	TAA.ti,ab.	9,987
14	aTAA.ti,ab.	269
15	thoracic aort* aneurysm*.ti,ab.	6,130
16	ascending aort* aneurysm*.ti,ab.	2,365
17	(aort* adj3 dissection*).ti,ab.	30,583
18	(aort* adj3 aneurysm*).ti,ab.	62,689
19	(dissect* adj3 aneurysm*).ti,ab.	12,356
20	aort* root.ti,ab.	19,107
21	1 or 2 or 3 or 4 or 5 or 6 or 7 or 8 or 9 or 10 or 11 or 12 or 13 or 14 or 15 or 16 or 17 or 18 or 19 or 20	171,375
22	growth rate/	62,013

23	growth rate*.ti,ab.	96,935
24	dilat* rate*.ti,ab.	475
25	progression rate*.ti,ab.	9,463
26	expansion rate*.ti,ab.	2,000
27	millimeter progression.ti,ab.	1
28	diameter progression.ti,ab.	60
29	(natural history and aneurysm*).ti,ab.	2,393
30	22 or 23 or 24 or 25 or 26 or 27 or 28 or 29	132,284
31	21 and 30	2,184

S1.4 Comprehensive List of Excluded Studies

Seventy-three studies were reviewed in full text. The list of excluded (n=70) studies and reason for exclusion is provided in the table S1.4.1 below. Of these 70 studies we found 11 of high relevance, with potentially eligible and valuable information on sex-specific aTAA growth, but unfortunately the data was reported in a disaggregated form, or they included bicuspid aortic valves or syndromic patients leading to exclusion due to wrong study population. The 11 studies of high relevance are marked in **bold**. Corresponding authors of these 11 studies were contacted on two separate occasions in an attempt to obtain relevant unpublished results or data for further analysis. This resulted in the inclusion of two studies; Fleury et al. and Viitala et al., reducing the total number of excluded studies to n = 68.

Table S1.4.1 Comprehensive List of Studies Excluded in the Initial Database/Register Search

N.	Title	Authors	Year	Reason for exclusion
1	Thoracic aortic disease: why sex matters	Dumfarth J., et al.	2025	Exclusion reason: Wrong study design;

Table S1.4.1 Comprehensive List of Studies Excluded in the Initial Database/Register Search

2	Thoracic Aortic Aneurysm Growth Rates and Predicting Factors: A Systematic Review and Meta-Analysis.	Henry M., et al.	2025	Exclusion reason: Wrong study design;
3	Ethnic Differences in Ascending Aorta Dimensions and Dilatation Rates: A Systematic Review.	Ansari A., et al.	2024	Exclusion reason: Wrong study design;
4	Growth rates in non-syndromic aneurysms of the ascending aorta: a systematic review.	Cozijnsen L., et al.	2024	Exclusion reason: Wrong study design;
5	Systematic Review of the Growth Rates and Influencing Factors in Thoracic Aortic Aneurysms.	Oladokun D., et al.	2016	Exclusion reason: Wrong study design;
6	[Aneurysms of the ascending aorta and aortic arch].	Leontyev S., et al.	2014	Exclusion reason: Wrong study design;
7	How robust are the natural history data of ascending aortic aneurysm? A systematic review and failed meta-analysis	Guo M., et al.	2016	Exclusion reason: Wrong study design;
8	Ascending Aortic Aneurysm	Gunn, T.M., et al.	2019	Exclusion reason: Wrong study design;
**9**	**Sex-related differences in the clinical course of aortic root and ascending aortic aneurysms: the DisSEXion Study.**	**Notenboom M.L., et al.**	**2025**	**Exclusion reason: Wrong patient population;**
**10**	**Effect of aortic valve phenotype and sex on aorta dilation in patients with aortic stenosis.**	**Fleury M. A., et al.**	**2024**	**Exclusion reason: Wrong patient population; included after author contact**
**11**	**Sex differences in ascending aortic size reporting and growth on chest computed tomography and magnetic resonance imaging.**	**Zamirpour S., et al.**	**2024**	**Exclusion reason: Wrong patient population;**
**12**	**Fate of the unoperated ascending thoracic aortic aneurysm: three-decade experience from the Aortic Institute at Yale University.**	**Wu J., et al.**	**2023**	**Exclusion reason: Wrong patient population;**
13	Ascending thoracic aortic aneurysm growth is minimal at sizes that do not meet criteria for surgical repair.	Gulati A., et al.	2022	Exclusion reason: Wrong patient population;
**14**	**Diameter and growth rate of the thoracic aorta-analysis based on serial computed tomography scans.**	**Chang H.W., et al.**	**2020**	**Exclusion reason: Wrong patient population;**

Table S1.4.1 Comprehensive List of Studies Excluded in the Initial Database/Register Search

15	Growth of the thoracic aorta in the smoking population: The Danish Lung Cancer Screening Trial.	Bons L.R., et al.	2020	Exclusion reason: Wrong patient population;
**16**	**Sex Differences in Thoracic Aortic Aneurysm Growth.**	**Boczar K.E., et al.**	**2019**	**Exclusion reason: Wrong patient population;**
17	Aortic aneurysms: growth rates measured with CT.	Hirose Y., et al.	1992	Exclusion reason: Wrong patient population;
18	Male-female specific aortic growth after 10 year follow-up in an aged population	Thijssen C., et al.	2020	Exclusion reason: Wrong patient population;
19	Rate of Ascending Aortic Enlargement in a Large Echocardiographic Cohort: Associated Risk Factors and Adverse Aortic Events.	Westenfield K., et al.	2025	Exclusion reason: No sex stratification; no access;
**20**	**Follow-up of incidentally detected mild to moderate ascending aortic dilation and risk factors for rapid progression in a Swedish middle-aged population.**	**Kylhammar D., et al.**	**2025**	**Exclusion reason: No sex stratification;**
21	Three-dimensional assessment of ascending aortic stiffness, motion, and growth in ascending thoracic aortic aneurysm.	Tjahjadi N. S., et al.	2025	Exclusion reason: Wrong study design;
22	Surgery versus surveillance for ascending aortic aneurysms in elderly patients.	Chan V. F., et al.	2024	Exclusion reason: No sex stratification;
**23**	**Linear growth pattern can be used to predict ascending aortic aneurysm growth.**	**Viitala I. M., et al.**	**2024**	**Exclusion reason: No sex stratification; included after author contact**
24	Aortic events and relative survival in patients with moderately dilated proximal thoracic aorta.	Carlestål E., et al.	2024	Exclusion reason: No sex stratification;
**25**	**Baseline Diameter Does Not Predict Growth Rate in a Presurgical Ascending Thoracic Aortic Aneurysm Population.**	**Marway P. S., et al.**	**2024**	**Exclusion reason: No sex stratification;**
26	Bicuspid aortopathy does not require earlier surgical intervention.	Zafar M. A., et al.	2024	Exclusion reason: No sex stratification;
27	Differential expansion and outcomes of ascending and descending degenerative thoracic aortic aneurysms.	Huang Y., et al.	2024	Exclusion reason: No sex stratification;

Table S1.4.1 Comprehensive List of Studies Excluded in the Initial Database/Register Search

28	Ascending thoracic aortic aneurysm size at presentation and growth by diameter.	Pace W. A., et al.	2023	Exclusion reason: No sex stratification;
**29**	**Ascending Aortic Aneurysms <4.5 cm for Nonsyndromic Adults: Very Slow Growth and Low Risk.**	**Hiratzka L. F., et al.**	**2023**	**Exclusion reason: No sex stratification;**
**30**	**Growth rate of ascending thoracic aortic aneurysms in a non-referral-based population.**	**Weininger G., et al.**	**2022**	**Exclusion reason: No sex stratification;**
31	Central Hypertension in Patients With Thoracic Aortic Aneurysms: Prevalence and Association With Aneurysm Size and Growth.	Rooprai J., et al.	2022	Exclusion reason: No sex stratification;
**32**	Determining the optimal interval for imaging surveillance of ascending aortic aneurysms.	Adriaans B. P., et al.	2021	Exclusion reason: No sex stratification;
33	Patterns of ascending aortic dilatation and predictors of surgical replacement of the aorta: A comparison of bicuspid and tricuspid aortic valve patients over eight years of follow-up.	Agnese V., et al.	2019	Exclusion reason: No sex stratification;
34	Height alone, rather than body surface area, suffices for risk estimation in ascending aortic aneurysm.	Zafar M.A., et al.	2018	Exclusion reason: No sex stratification;
35	Natural history of moderately dilated tubular ascending aorta: implications for determining the optimal imaging interval.	Park K.H., et al.	2017	Exclusion reason: No sex stratification;
36	Natural history of 40-50 mm root/ascending aortic aneurysms in the current era of dedicated thoracic aortic clinics.	Gagné-Loranger M., et al.	2016	Exclusion reason: No sex stratification;
37	Natural history and risk factors for rupture of thoracic aortic arch aneurysms.	Yiu R.S., et al.	2016	Exclusion reason: No sex stratification;
38	Effects of statin therapy on ascending aorta aneurysms growth: A propensity-matched analysis.	Angeloni E., et al.	2015	Exclusion reason: No sex stratification;
39	Surveillance of moderate-size aneurysms of the thoracic aorta.	McLarty A.J., et al.	2015	Exclusion reason: No sex stratification;
40	Prevalence and significance of incidentally noted dilation of the ascending aorta on routine chest computed tomography in older patients.	Benedetti N., et al.	2015	Exclusion reason: No sex stratification;
41	A prospective study of growth and rupture risk of small-to-moderate size ascending aortic aneurysms.	Geisbüsch S., et al.	2014	Exclusion reason: No sex stratification;

Table S1.4.1 Comprehensive List of Studies Excluded in the Initial Database/Register Search

42	Progression rate of ascending aortic dilation in patients with normally functioning bicuspid and tricuspid aortic valves.	La Canna G., et al.	2006	Exclusion reason: No sex stratification;
43	Natural history of thoracic aortic aneurysms: indications for surgery, and surgical versus nonsurgical risks.	Elefteriades J.A.	2002	Exclusion reason: No sex stratification;
44	Prediction of thoracic aortic aneurysm expansion: validation of formulae describing growth.	Shimada I., et al.	1999	Exclusion reason: No sex stratification;
45	Ascending Thoracic Aortic Aneurysm Growth Rate Stratified by Size at Presentation	Pace W.A., et al.	2023	Exclusion reason: No sex stratification;
46	Single vs Serial Assessments of Arterial Hemodynamics to Predict Thoracic Aortic Aneurysm Expansion	Mian O., et al.	2021	Exclusion reason: No sex stratification;
47	Accurate and reproducible aortic growth rate mapping via registration of serial contrast-enhanced computed tomography angiograms	Dux-Santoy L., et al.	2021	Exclusion reason: No sex stratification;
48	Aortic elasticity indexes by magnetic resonance predict progression of ascending aorta dilation	Aquaro G.D., et al.	2016	Exclusion reason: No sex stratification;
49	Multimodality serial follow-up of thoracic aortic aneurysms	Rizzo J.A., et al.	1997	Exclusion reason: No sex stratification;
50	Ascending aorta dilatation rates in patients with tricuspid and bicuspid aortic stenosis: The COFRASA/GENERAC study	Kerneis C., et al.	2018	Exclusion reason: No sex stratification;
51	Risk of Aortic Dissection in the Moderately Dilated Ascending Aorta	Kim, J. B., et al.	2016	Exclusion reason: No sex stratification;
52	Aortic Stiffness, Central Blood Pressure, and Pulsatile Arterial Load Predict Future Thoracic Aortic Aneurysm Expansion.	Boczar K. E., et al.	2021	Exclusion reason: No sex stratification;
53	INFLUENCE OF AORTIC VALVE PHENOTYPE, MORPHOLOGY AND SEX ON ASCENDING AORTA DILATION	Fleury M., et al.	2023	Exclusion reason: Abstract;Full article 2024
54	GENDER DISPARITIES IN THE NATURAL HISTORY OF ASCENDING THORACIC AORTIC ANEURYSM: SYSTEMATIC REVIEW AND META-ANALYSIS	Ubaid M., et al.	2025	Exclusion reason: Abstract; no access;
55	Monitoring Progression of Aneurysms of the Ascending Aorta: Timing, Cost and Radiation Exposure	Callow A.E., et al.	2020	Exclusion reason: Abstract; no access;

Table S1.4.1 Comprehensive List of Studies Excluded in the Initial Database/Register Search

56	The natural history of Thoracic Aortic Aneurysm(TAA) expansion	Shimada, I., et al.	1998	Exclusion reason: Abstract; no access;
57	Natural History of Ascending Aortic Aneurysms with Bicuspid and Tricuspid Aortic Valves	Tharakan S., et al.	2020	Exclusion reason: Abstract; full text 2022;
58	Central hypertension in apparently normotensive patients with thoracic aortic aneurysms: Prevalence and association with aneurysm expansion	Rooprai J., et al.	2019	Exclusion reason: Abstract; Article in 2022;
59	Natural history of medically treated ascending aortic aneurysms in the era of thoracic aortic clinics: Impact of bicuspid aortic valve and clinical implications	Dagenais F., et al.	2016	Exclusion reason: Abstract;
60	Baseline Diameter Does Not Predict Growth Rate in Ascending Thoracic Aortic Aneurysm	Marway P.S., et al.	2023	Exclusion reason: Abstract;
61	TIME-UPDATED ANALYSIS OF THORACIC AORTIC ANEURYSM SIZE TO ESTIMATE LONG-TERM RISKS OF AORTIC DISSECTION	Solomon M.D., et al.	2022	Exclusion reason: Abstract;
62	431 Very Long Term Follow-Up (>3000 Days) of Aortopathy Patients - Surgical Intervention/Dissection is Predicted by Accelerated Annualised Aortic Growth Rate	Humphries J., et al.	2020	Exclusion reason: Abstract;
63	MEASURES OF AORTIC STIFFNESS, CENTRAL BLOOD PRESSURE AND PULSATILE ARTERIAL LOAD PREDICT FUTURE ANEURYSM EXPANSION	Boczar K., et al.	2019	Exclusion reason: Abstract;
64	Sex Differences in the Growth Rates of Thoracic Aortic Aneurysms: Role of Aneurysm Etiology	Cheung K., et al.	2016	Exclusion reason: Abstract;
65	Identifying dilated aortic root diameters in Nova Scotia; is a screening program warranted?	Herman C., et al.	2013	Exclusion reason: Abstract;
66	Effect of Presenting Size on Sex Differences in Ascending Aortic Growth Rate	Zamirpour S., et al.	2023	Exclusion reason: Abstract;
67	Patient Characteristics, Clinical Presentation, Location, and Outcomes of Saccular Thoracic Aortic Aneurysms in the Vascular Quality Initiative	Rastogi V.; et al.	2022	Exclusion reason: Abstract;
68	Sex differences in thoracic aortic aneurysm growth: Role of aortic stiffness	Coutinho T., et al.	2017	Exclusion reason: Abstract;
69	Sex differences in growth rates of thoracic aortic aneurysms	Cheung K., et al.	2015	Exclusion reason: Abstract;Full article in 2017
70	Upper limit of expansion rates of thoracic aortic diameter in asymptomatic population evaluated by a routine heart CT scan	Mao S.S., et al.	2015	Exclusion reason: Abstract;

S2 Quality and Risk of Bias Evaluation of Included Studies

S2.1 Description of the Quality of Studies Evaluation (QoS) Tool

The Quality of Studies (QoS) Evaluation Tool was developed by authors (HMP, RMJG) to assess the primary objective of this systematic review: the growth rates of the aortic aneurysms measured by imaging techniques. Upon review of existing validated tools inspiration was drawn from tools such as Cochrane Risk Of Bias In Non-randomized Studies – of Interventions, Version 2 (ROBINS-I V2) and the Methodological Index for Non-Randomized Studies (MINORS). Items 5, 6, and 7 were adapted from MINORS and the corresponding MINORS criteria is shown in parentheses next to item in question.

As with any quality or bias evaluation tool, a low QoS score should be taken into consideration when interpreting a study's findings. Accordingly, as outlined in Table S1, the total score assigned to each study results in the following grading of quality: poor, moderate, or good. We advise that growth rates reported in studies rated as poor should be interpreted with caution.

The detailed description of the scoring criteria for each of the seven items included in the QoS tool is provided below. Element 1-4 evaluates the methodology whereas element 5-7 assesses the of risk of bias linked to obtaining and presenting results. The Quality of Studies (QoS) assessment was conducted independently by two authors (RMJG and HMP). No discrepancies in scoring were identified.

S2.2 Definition of Each Item and Scoring Rationale

1. **Is the definition of the ascending thoracic aortic aneurysm based on a personalized, indexed aortic diameter?**
   0 = A personalized, indexed aortic diameter is not taken into consideration when defining, analyzing, or discussing aneurysms; 0.5 = Personalized or indexed definitions are mentioned but are incompletely described, or they are not incorporated into the analysis or discussion of results; 1 = A clearly described personalized, indexed definition is used or explicitly referenced. The indexed definition is taken into consideration when analyzing or discussing results.
2. **Was the imaging method standardized and clearly described?**
   0 = Imagining method not described; 0.5 = Described insufficiently or not standardized for each patient 1 = Clearly described and standardized, sufficient for replication of study. Factors considered included the use of concordant imagining technique during follow ups, a specialist performing measurements or validation of measuring, mentioning model of imagining equipment or stating slice thickness when CT was used.
3. **Is the measurement technique described?**
   0 = Not described; 0.5 = Described insufficiently; 1 = Clear description of measurement technique used, sufficient for replication of study. Factors considered included whether the study specified the training of the individual performing the measurements, which procedure or guideline was followed when measuring the aorta (e.g., inner-to-inner diameter), and whether the calculation of growth rate was clearly described.
4. **Were there >2 measurement time points?**

It is important to note the requirement of more than two time points at which imaging scans were performed. 0 = ≤2 time points; 1 = more than two time points.

5. **Was the follow-up period sufficient, ≥1 year? (MINORS, 6)**
   0 = <1 year or unclearly reported; 1 = ≥1 year and clearly described.
6. **Was loss during follow-up clearly described with <5% unexplained missing data? (MINORS, 7)**
   0 = Not reported or unclear; 0.5 = Reported but loss during follow-up >5%; 1 = Reported and <5%
7. **Were confidence intervals and / or statistical significance reported in the original article? (MINORS, 8)**
   0 = No confidence intervals or significance reported; 0.5 = confidence intervals or significance reported for the main analysis; 1 = Clearly reported confidence intervals or significance for all relevant analyses related to results and conclusion.

Table S2.1 Cut-off Points for QoS Grades

Score Range	Grade	Justification
0.0 - 2.5	Poor	Lacks compliance with standardized definitions and methods. A low reliability or possible bias in aneurysm measurement is likely.
3.0 - 5.0	Moderate	Standardized definitions and methods are used to some degree. A low reliability or possible bias in aneurysm measurement cannot be ruled out.
5.5 - 7.0	Good	Good compliance with imaging and reporting standards which results in high reliability and reproducibility.

S3 Completed PRISMA 2020 Guideline Checklists

S3.1 PRISMA 2020 Abstracts Checklist

PRISMA 2020 for Abstracts Checklist

Section and Topic	Item #	Checklist item	Reported (Yes/No)
**TITLE**			
Title	1	Identify the report as a systematic review.	Yes
**BACKGROUND**			
Objectives	2	Provide an explicit statement of the main objective(s) or question(s) the review addresses.	Yes
**METHODS**			
Eligibility criteria	3	Specify the inclusion and exclusion criteria for the review.	Yes
Information sources	4	Specify the information sources (e.g. databases, registers) used to identify studies and the date when each was last searched.	Yes
Risk of bias	5	Specify the methods used to assess risk of bias in the included studies.	No
Synthesis of results	6	Specify the methods used to present and synthesise results.	No
**RESULTS**			
Included studies	7	Give the total number of included studies and participants and summarise relevant characteristics of studies.	No
Synthesis of results	8	Present results for main outcomes, preferably indicating the number of included studies and participants for each. If meta-analysis was done, report the summary estimate and confidence/credible interval. If comparing groups, indicate the direction of the effect (i.e. which group is favoured).	Yes
**DISCUSSION**			
Limitations of evidence	9	Provide a brief summary of the limitations of the evidence included in the review (e.g. study risk of bias, inconsistency and imprecision).	No
Interpretation	10	Provide a general interpretation of the results and important implications.	Yes
**OTHER**			
Funding	11	Specify the primary source of funding for the review.	No
Registration	12	Provide the register name and registration number.	Yes

From: Page MJ, McKenzie JE, Bossuyt PM, Boutron I, Hoffmann TC, Mulrow CD, et al. The PRISMA 2020 statement: an updated guideline for reporting systematic reviews. BMJ 2021;372:n71. doi: 10.1136/bmj.n71. This work is licensed under CC BY 4.0. To view a copy of this license, visit https://creativecommons.org/licenses/by/4.0/

S3.2 PRISMA Comprehensive Checklist

PRISMA 2020 Checklist

Section and Topic	Item #	Checklist item	Location where item is reported
**TITLE**			
Title	1	Identify the report as a systematic review.	1
**ABSTRACT**			
Abstract	2	See the PRISMA 2020 for Abstracts checklist.	Supplementary Materials S3.1
**INTRODUCTION**			
Rationale	3	Describe the rationale for the review in the context of existing knowledge.	2
Objectives	4	Provide an explicit statement of the objective(s) or question(s) the review addresses.	2
**METHODS**			
Eligibility criteria	5	Specify the inclusion and exclusion criteria for the review and how studies were grouped for the syntheses.	3-4
Information sources	6	Specify all databases, registers, websites, organisations, reference lists and other sources searched or consulted to identify studies. Specify the date when each source was last searched or consulted.	3-4, Supplementary Materials S1.1, S.1.3
Search strategy	7	Present the full search strategies for all databases, registers and websites, including any filters and limits used.	2-5, Supplementary materials S1.1, S1.2, S1.3.
Selection process	8	Specify the methods used to decide whether a study met the inclusion criteria of the review, including how many reviewers screened each record and each report retrieved, whether they worked independently, and if applicable, details of automation tools used in the process.	3-6, Figure 2
Data collection process	9	Specify the methods used to collect data from reports, including how many reviewers collected data from each report, whether they worked independently, any processes for obtaining or confirming data from study investigators, and if applicable, details of automation tools used in the process.	3-4, Figure 2
Data items	10a	List and define all outcomes for which data were sought. Specify whether all results that were compatible with each outcome domain in each study were sought (e.g. for all measures, time points, analyses), and if not, the methods used to decide which results to collect.	4-10, Table 3
	10b	List and define all other variables for which data were sought (e.g. participant and intervention characteristics, funding sources). Describe any assumptions made about any missing or unclear information.	6, 9-10, Table 1, Table 2, Supplementary Materials S2.1, S.2.2, Table S2.1
Study risk of bias assessment	11	Specify the methods used to assess risk of bias in the included studies, including details of the tool(s) used, how many reviewers assessed each study and whether they worked independently, and if applicable, details of automation tools used in the process.	9-10, Supplementary Materials S2
Effect measures	12	Specify for each outcome the effect measure(s) (e.g. risk ratio, mean difference) used in the synthesis or presentation of results.	8-9, Figure 3
Synthesis methods	13a	Describe the processes used to decide which studies were eligible for each synthesis (e.g. tabulating the study intervention characteristics and comparing against the planned groups for each synthesis (item #5)).	3-5, 10-12
	13b	Describe any methods required to prepare the data for presentation or synthesis, such as handling of missing summary statistics, or data conversions.	3-4, Table 2, Table 3
	13c	Describe any methods used to tabulate or visually display results of individual studies and syntheses.	4
	13d	Describe any methods used to synthesize results and provide a rationale for the choice(s). If meta-analysis was performed, describe the	4, Figure 3

PRISMA 2020 Checklist

Section and Topic	Item #	Checklist item	Location where item is reported
		model(s), method(s) to identify the presence and extent of statistical heterogeneity, and software package(s) used.	
	13e	Describe any methods used to explore possible causes of heterogeneity among study results (e.g. subgroup analysis, meta-regression).	10-12
	13f	Describe any sensitivity analyses conducted to assess robustness of the synthesized results.	None performed
Reporting bias assessment	14	Describe any methods used to assess risk of bias due to missing results in a synthesis (arising from reporting biases).	Supplementary Materials S2.2 item 6
Certainty assessment	15	Describe any methods used to assess certainty (or confidence) in the body of evidence for an outcome.	9-10, Supplementary Materials S2
**RESULTS**			
Study selection	16a	Describe the results of the search and selection process, from the number of records identified in the search to the number of studies included in the review, ideally using a flow diagram.	3, 5, Figure 2
	16b	Cite studies that might appear to meet the inclusion criteria, but which were excluded, and explain why they were excluded.	3-4, Supplementary Materials S1.4
Study characteristics	17	Cite each included study and present its characteristics.	6, Table 1
Risk of bias in studies	18	Present assessments of risk of bias for each included study.	9-10, Supplementary Materials S2
Results of individual studies	19	For all outcomes, present, for each study: (a) summary statistics for each group (where appropriate) and (b) an effect estimate and its precision (e.g. confidence/credible interval), ideally using structured tables or plots.	8, Table 3
Results of syntheses	20a	For each synthesis, briefly summarise the characteristics and risk of bias among contributing studies.	10, Table 4, 12
	20b	Present results of all statistical syntheses conducted. If meta-analysis was done, present for each the summary estimate and its precision (e.g. confidence/credible interval) and measures of statistical heterogeneity. If comparing groups, describe the direction of the effect.	9, Figure 3
	20c	Present results of all investigations of possible causes of heterogeneity among study results.	10-12
	20d	Present results of all sensitivity analyses conducted to assess the robustness of the synthesized results.	None performed.
Reporting biases	21	Present assessments of risk of bias due to missing results (arising from reporting biases) for each synthesis assessed.	10, Table 4
Certainty of evidence	22	Present assessments of certainty (or confidence) in the body of evidence for each outcome assessed.	10, Table 4, Supplementary Materials S2
**DISCUSSION**			
Discussion	23a	Provide a general interpretation of the results in the context of other evidence.	10-12
	23b	Discuss any limitations of the evidence included in the review.	10-13
	23c	Discuss any limitations of the review processes used.	10-13
	23d	Discuss implications of the results for practice, policy, and future research.	12
**OTHER INFORMATION**			
Registration and	24a	Provide registration information for the review, including register name and registration number, or state that the review was not	1

PRISMA 2020 Checklist

Section and Topic	Item #	Checklist item	Location where item is reported
protocol		registered.	
	24b	Indicate where the review protocol can be accessed, or state that a protocol was not prepared.	1
	24c	Describe and explain any amendments to information provided at registration or in the protocol.	PROSPERO CRD420251025890, see version history
Support	25	Describe sources of financial or non-financial support for the review, and the role of the funders or sponsors in the review.	12
Competing interests	26	Declare any competing interests of review authors.	13
Availability of data, code and other materials	27	Report which of the following are publicly available and where they can be found: template data collection forms; data extracted from included studies; data used for all analyses; analytic code; any other materials used in the review.	13

From: Page MJ, McKenzie JE, Bossuyt PM, Boutron I, Hoffmann TC, Mulrow CD, et al. The PRISMA 2020 statement: an updated guideline for reporting systematic reviews. BMJ 2021;372:n71. doi: 10.1136/bmj.n71.
This work is licensed under CC BY 4.0. To view a copy of this license, visit https://creativecommons.org/licenses/by/4.0/

Reference List for Supplementary Materials

1. Slim K, Nini E, Forestier D, Kwiatkowski F, Panis Y, Chipponi J. Methodological index for non-randomized studies (minors): development and validation of a new instrument. *ANZ J Surg*. Sep 2003;73(9):712-6. doi:10.1046/j.1445-2197.2003.02748.x
